# MiR-338-3p inhibits epithelial-mesenchymal transition in gastric cancer cells by targeting ZEB2 and MACC1/Met/Akt signaling

**DOI:** 10.18632/oncotarget.3835

**Published:** 2015-04-14

**Authors:** Na Huang, Zhenzhen Wu, Li Lin, Minyu Zhou, Lin Wang, Huanrong Ma, Jianling Xia, Jianping Bin, Yulin Liao, Wangjun Liao

**Affiliations:** ^1^ Department of Oncology, Nanfang Hospital, Southern Medical University, Guangzhou, China; ^2^ Department of Cardiology, Nanfang Hospital, Southern Medical University, Guangzhou, China

**Keywords:** gastric cancer, miR-338-3p, ZEB2, MACC1, epithelial-mesenchymal transition

## Abstract

MicroRNAs (miRNAs) are involved in the epithelial-mesenchymal transition (EMT) process and are associated with metastasis in gastric cancer (GC). MiR-338-3p has been reported to be aberrantly expressed in GC. In the present study, we show that miR-338-3p inhibited the migration and invasion of GC cells *in vitro*. Knocking down miR-338-3p in GC cells led to mesenchymal-like changes. MiR-338-3p influenced the expression of the EMT-associated proteins by upregulating the epithelial marker E-cadherin and downregulating the mesenchymal markers, N-cadherin, fibronectin, and vimentin. In terms of mechanism, miR-338-3p directly targeted zinc finger E-box-binding protein 2 (ZEB2) and metastasis-associated in colon cancer-1 (MACC1). MiR-338-3p repressed the Met/Akt pathway after MACC1 inhibition. Reintroduction of ZEB2 and MACC1 reversed miR-338-3p-induced EMT suppression. Consistently, inverse correlations were also observed between the expression of miR-338-3p and ZEB2 or MACC1 in human GC tissue samples. In conclusion, miR-338-3p inhibited the EMT progression in GC cells by targeting ZEB2 and MACC1/Met/Akt signaling.

## INTRODUCTION

The epithelial-mesenchymal transition (EMT) is considered as one of critical steps in gastric cancer (GC) cell invasion and metastasis, by which GC cells lose their epithelial phenotype and cell-cell adhesion and gain invasive mesenchymal properties [1-3]. The molecular mechanisms of EMT are intricate. Certain transcription factors such as zinc finger E-box-binding protein (ZEB) are involved in EMT regulation in GC [3-5]. Several signaling pathways may also contribute to EMT regulation. For example, the Met/Akt pathway has been previously determined to promote EMT in GC [6, 7].

Recent studies have shown that microRNAs (miRNAs) via binding to the 3′ untranslated region (UTR) of some target genes cause the mRNA destabilization and protein downregulation, which may result in the repression of the EMT process [8-10]. MiR-338-3p has been reported to function as a tumor suppressor gene by inhibiting the invasion of hepatocellular carcinoma [11]. A recent study showed that miR-338-3p is lower in GC tissues than normal tissues [9]. Moreover, GC miR-338-3p expression is even lower in patients with deeper local invasion and advanced TNM stage [9]. These not only suggest that miR-338-3p is a biomarker of GC, but might also be involved in EMT regulation that initiates GC cell invasion. However, the underlying mechanism of this process has not been well explored.

One of the identified mechanisms of the EMT involves miRNAs directly acting on specific EMT-associated transcription factors. For example, the miR-200 family suppresses EMT by targeting ZEB1 and ZEB2 [8,12]. ZEB2, also known as smad-interacting protein 1 (SIP1), is a member of the zinc finger E-box-binding protein (ZEB) family and has been reported to play an important role in inducing the EMT in GC [5,13]. Bioinformatics analysis has indicated that ZEB2 is a putative target of miR-338-3p. However, the association of miR-338-3p and ZEB2 has not been investigated in GC. In addition, miRNAs may regulate several EMT components and alter the expression of certain oncogenes or tumor suppression genes [14]. Recently, we have discovered that metastasis-associated in colon cancer-1 (MACC1) participates in GC progression. Upregulating MACC1, the upstream modulating factor of Met, can also enhance the EMT process in GC cells, whereas silencing MACC1 inhibits GC cell invasion and metastasis [15]. Previous study showed MACC1 expression can be suppressed by miR-143 in colon cancer cells [16]. However, whether any other miRNAs participated in GC MACC1 regulation remains uncertain.

Based on previous findings, we assumed that miR-338-3p might be involved in the EMT of GC cells, and ZEB2 or MACC1 might be involved in this process. Accordingly, we performed quantitative real-time polymerase chain reaction (qRT-PCR) analysis to detect miR-338-3p level in GC tissue samples and cell lines, and conducted wound healing, transwell, three-dimensional (3D) cell culture, and western blotting assays on transfected GC cell lines to explore the role of miR-338-3p in EMT. Dual-luciferase assays were conducted to verify whether miR-338-3p directly targeted MACC1 or any other EMT-correlated genes.

## RESULTS

### GC is deficient in miR-338-3p

**Figure 1:**
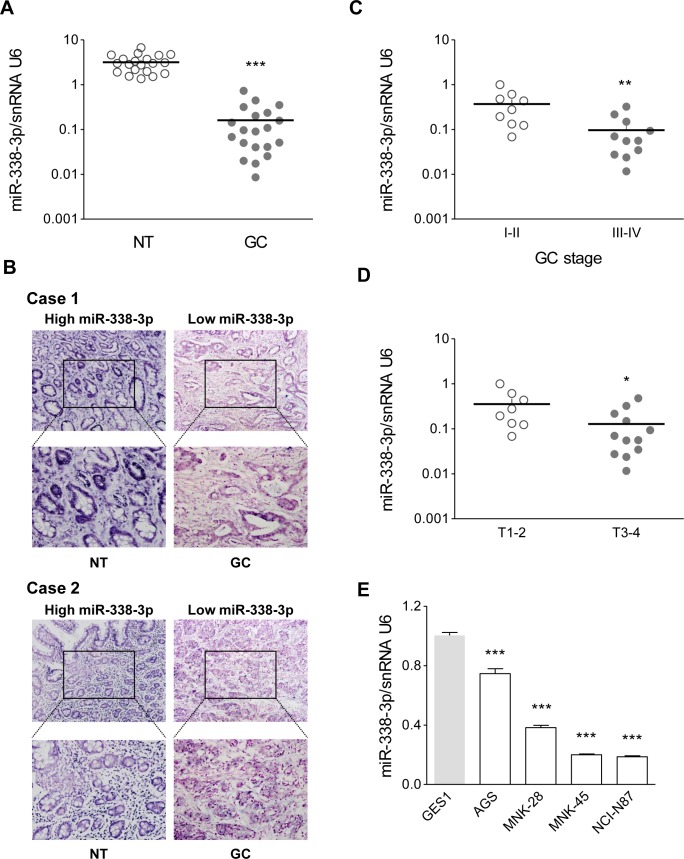
MiR-338-3p is expressed at low levels in advanced stages of GC (**A**) As analyzed by qRT-PCR, MiR-338-3p expression level in GC tissues was significantly lower than that observed in the corresponding noncancerous gastric mucosa tissue. (**B**) Representative images of miR-338-3p expression by ISH. (**C**) In GC tissues, the miR-338-3p level in advanced (stages III-IV) GC was lower than that in early (stages I-II) GC. (**D**) In GC tissues, miR-338-3p level T3-4 GC was lower than that observed in T1-2 samples. (**E**) The relative levels of miR-338-3p in GC cells (NCI-N87, AGS, MKN-28, and MKN-45) were evaluated by qRT-PCR. The gastric mucosa epithelial cell line GES-1 was used as control. *P < 0.05, **P < 0.01 and ***P < 0.001.

To confirm miR-338-3p expression in GC, qRT-PCR was performed in 20 pairs of human GC and their adjacent non-cancerous tissue samples. The expression of miR-338-3p was significantly lower in GC tissues than in the corresponding adjacent noncancerous tissues (Supplementary Table 3 and Figure 1A). Furthermore, in situ hybridization (ISH) showed that miR-338-3p was mainly localized in the cytoplasm of GC cells, with a certain amount detected in the cell nucleus (Figure 1B). Moreover, the mRNA expression of miR-338-3p was even lower in advanced stages (III-IV) of GC (n = 11) compared to that observed in the early stages (I-II) (n = 9) (Figure 1C, *P* = 0.007). In addition, miR-338-3p expression was lower in deeper invasion GC (T3-4 vs. T1-2), suggesting that its deficiency may contribute to GC cell invasiveness (Figure 1D, *P* = 0.031). Next, we analyzed miR-338-3p expression in different human cell lines. The expression of miR-338-3p in the GC cell lines, NCI-N87, AGS, MKN-28, and MKN-45 was significantly lower than that observed in the normal gastric mucosa cell line GES-1 (Figure 1E). This is consistent with our clinical findings that human GC tissues have low levels of miR-338-3p. In addition, the expression of miR-338-3p in AGS and MKN-28 cell lines was higher than MKN-45 and N87 cell lines. Therefore, we chose AGS (a poorly-differentiated cell line) and MKN-28 (a well-differentiated cell line) as representatives to perform the subsequent experiments.

### MiR-338-3p inhibits GC cell migration and invasion

**Figure 2:**
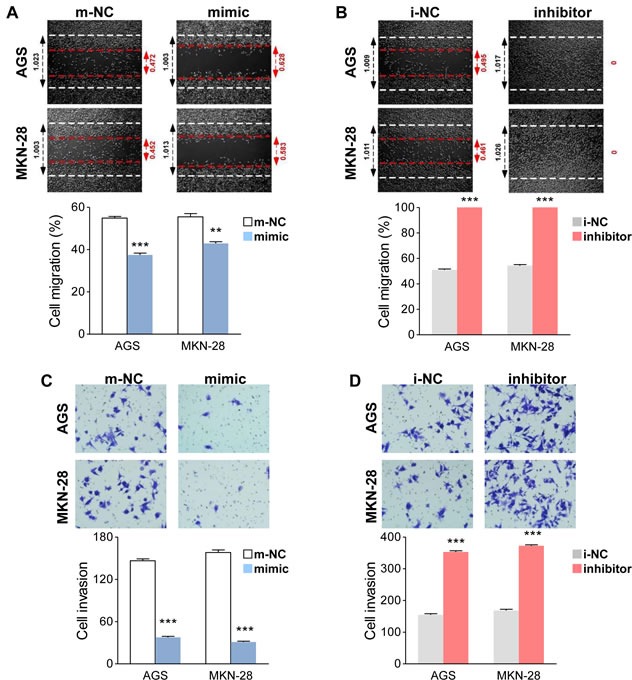
MiR-338-3p suppresses the motility and invasiveness of GC cells (**A** and **B**) Cell migration activity was measured by conducting the wound healing assay 48 h after transfection using miR-338-3p. (A) Restorated or (B) Downregulated cells. (**C** and **D**) For cellular invasiveness, transwell assays were performed on miR-338-3p (C) Restorated or (D) Downregulated cells.**P < 0.01 and ***P < 0.001.

The above clinical findings suggest miR-338-3p may participate in GC progression. Hence, we speculated that miR-338-3p might be associated with the migration and invasion of GC cells. To restore or downregulate miR-338-3p expression in GC cells, a miR-338-3p mimic and inhibitor were respectively transfected into AGS and MKN-28 cells. To investigate the potential effect of miR-338-3p on the motility and invasiveness, the wound healing and transwell invasive assays were performed in GC cells. The wound healing assay showed that the restoration of miR-338-3p significantly inhibited the migration capacity in both AGS and MKN-28 cells (Figure 2A). On the contrary, downregulating miR-338-3p expression using an inhibitor significantly enhanced the migratory activity of AGS and MKN-28 cells (Figure 2B). Meanwhile, the transwell invasive assay demonstrated that miR-338-3p restoration significantly repressed the invasiveness of AGS and MKN-28 cells, whereas inhibiting miR-338-3p expression facilitated GC cell invasion (Figures 2C and 2D). These results thus proved that miR-338-3p is a suppressor of migration and invasion in GC. Because EMT is one of the critical steps of tumor cell invasion, we then investigated whether miR-338-3p participates in the EMT process.

### MiR-338-3p inhibits the EMT process in GC cells

**Figure 3:**
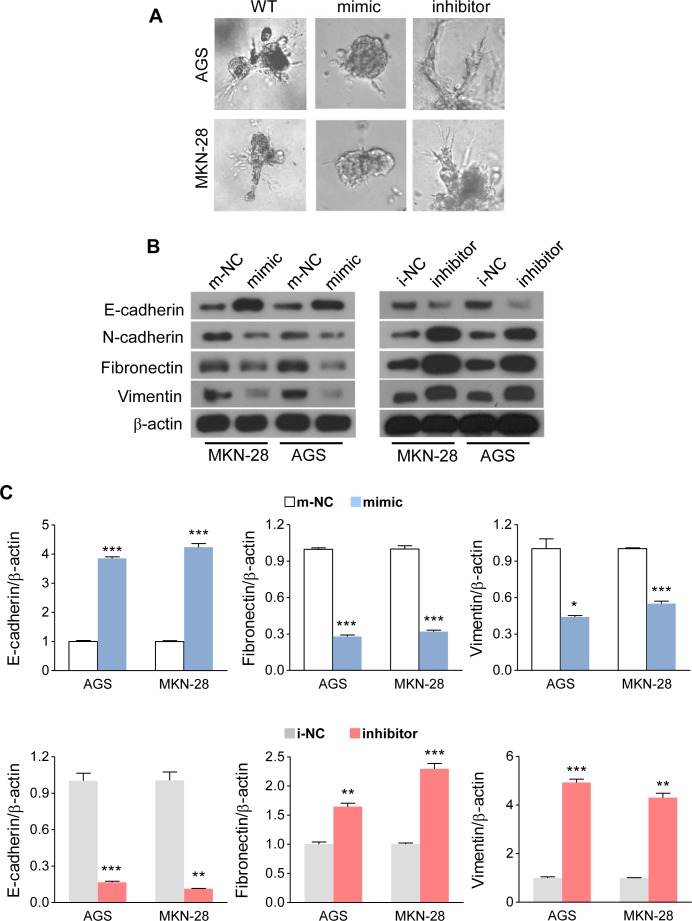
MiR-338-3p inhibits EMT in GC cells (**A**) Altering the miR-338-3p expression levels with mimic or inhibitor caused changes in the 3D morphology of AGS and MKN-28 cells. (B and C) After transfecting with miR-338-3p mimic or inhibitor, (**B**) the mRNA and (**C**) mRNA or protein expression levels of E-cadherin, N-cadherin, fibronectin and vimentin in AGS and MKN-28 cells were analyzed by (B) qRT-PCR or (C) Western blot. **P < 0.05, ** *P* < 0.01 and ***P < 0.001.

In the 3D culturing system, we observed that restoration of miR-338-3p transformed GC cells into spheroids with few protrusions, whereas suppressing miR-338-3p rendered GC cells to undergo spindle-like morphological changes (Figure 3A). This suggested that some molecular changes might have taken place during alterations in miR-338-3p expression. Next, we analyzed the mRNA and protein expression levels of an epithelial marker (E-cadherin) and several mesenchymal markers (N-cadherin, fibronectin and vimentin). Both western blot analysis (Figure 3B) and qRT-PCR (Figure 3C) revealed that the expressions of EMT-associated markers were altered by miR-338-3p restoration or inhibition. MiR-338-3p induction increased the level of the epithelial marker (E-cadherin) and suppressed the levels of the mesenchymal markers (N-cadherin, fibronectin, and vimentin). On the contrary, miR-338-3p inhibition demonstrated a completely reverse effect (Figures 3B and 3C). These results indicate that miR338-3p deficiency in GC cells might be an important contributor to EMT progression, thus facilitating the migration and invasion of GC cells.

### ZEB2 is a direct downstream target of miR-338-3p

**Figure 4:**
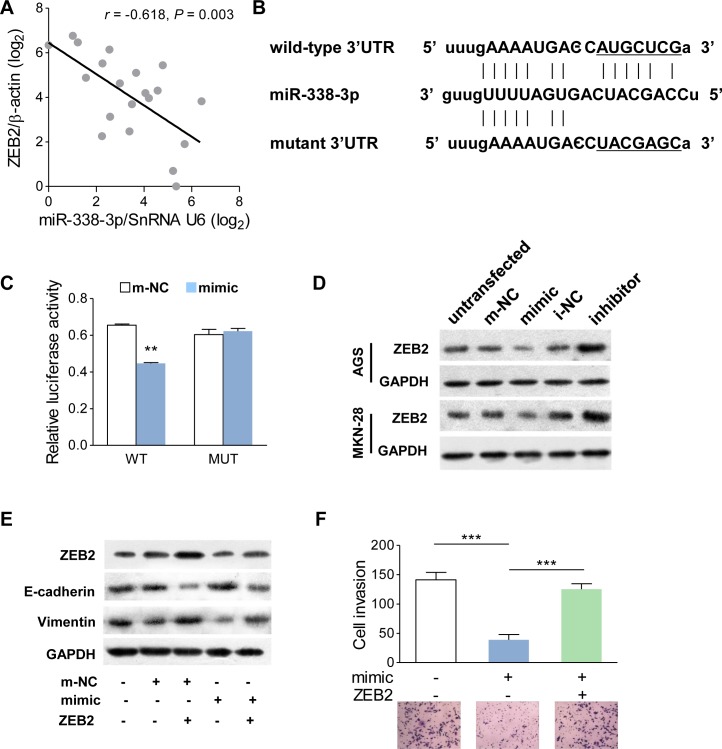
ZEB2 is a direct target of miR-338-3p (**A**) In human GC tissues, ZEB2 was negatively correlated with miR-338-3p at the mRNA level (n = 20). (**B**) The luciferase reporter of miR-338-3p binding site on the wild-type and mutant ZEB2 3′UTR. The replaced site is underlined. (**C**) Relative dual-luciferase activity analyses. The psiCHECK2-wZEB2 (WT) or psiCHECK2-mZEB2 (MUT) was cotransfected into HEK-293T cells with miR-338-3p mimic or its control plasmid. (**D**) Western blot analysis of ZEB2 protein in AGS and MKN-28 cells transfected with the miR-338-3p mimic or inhibitor. Untransfected cells as well as cells transfected with a control plasmid for mimic or inhibitor were used as controls. (**E**) The protein level of ZEB2, E-cadherin and vimentin was detected by western blot in cells transfected with ZEB2 expressing plasmid, miR-338-3p mimic, and/or its control plasmid. (**F**) Migratory activity was evaluated by transwell invasive assay in cells transfected with ZEB2-expressing plasmid and/or the miR-338-3p mimic. **P < 0.01 and ***P < 0.001.

It is generally believed that ZEB members are critical regulators of EMT modulation. In human GC tissues, we found that the mRNA level of ZEB2 was negatively correlated to miR-338-3p (Figure 4A), which suggested that a modulating effect existed between these two parameters. Bioinformatics analysis using miRanda (www.microrna.org) shown that the 3′UTR of ZEB2 is a binding site of miR-338-3p (Figure 4B). To confirm whether miR-338-3p suppressed EMT by targeting ZEB2, we initially constructed two types of plasmids containing the luciferase reporting gene and wild-type or mutant ZEB2 3′UTR and cotransfected a miR-338-3p mimic into HEK-293T cells (Figure 4C). GC cells co-transfected with a miR-338-3p mimic and wild-type ZEB2 3′UTR showed a significant decrease in luciferase activity (Figure 4C). However, in the mutant ZEB2 3′UTR group, no detectable change in luciferase activity was observed (Figure 4C), suggesting that miR-338-3p bind to ZEB2 3′UTR directly. Next, Western blot assay was performed to investigated whether the protein expression of ZEB2 was influenced. Compared to the control cells, the ZEB2 protein was down-regulated in cells with the miR-338-3p mimic but inversely upregulated in cells with the miR-338-3p inhibitor (Figure 4D). To determine whether miR-338-3p affected the EMT process by regulating ZEB2 expression, ZEB2 was reintroduced into miR-338-3p mimic-transfected AGS cells. Although miR-338-3p increased E-cadherin expression and decreased vimentin expression, ZEB2 induction significantly ameliorated these activities by suppressing E-cadherin and promoting vimentin expression (Figure 4E). These molecular changes also influenced GC cell function that miR-338-3p suppressed invasiveness was reversed by ZEB2 induction (Figure 4F). Taken together, these results showed that miR-338-3p deficiency upregulated ZEB2, which in turn mediated the EMT in GC.

Previous studies have shown that several miRNAs, such as miR-200 family can regulate ZEB2 expression, and induce EMT in GC [17,18]. Therefore, we also examined the mRNA levels of E-cadherin, N-cadherin, and vimentin in MKN-28 cells transfected with miR-200 family (miR-200a, miR-200b, miR-200c and miR-141) mimics or miR-338-3p mimics by qRT-PCR. As shown in Supplementary Figure 1, miR-200a, miR-200b, miR-200c, or miR-141 in combination with miR-338-3p can promote the epithelial phenotype in GC cells.

### MiR-338-3p represses MACC1/Met/Akt signaling and EMT by directly targeting MACC1

**Figure 5:**
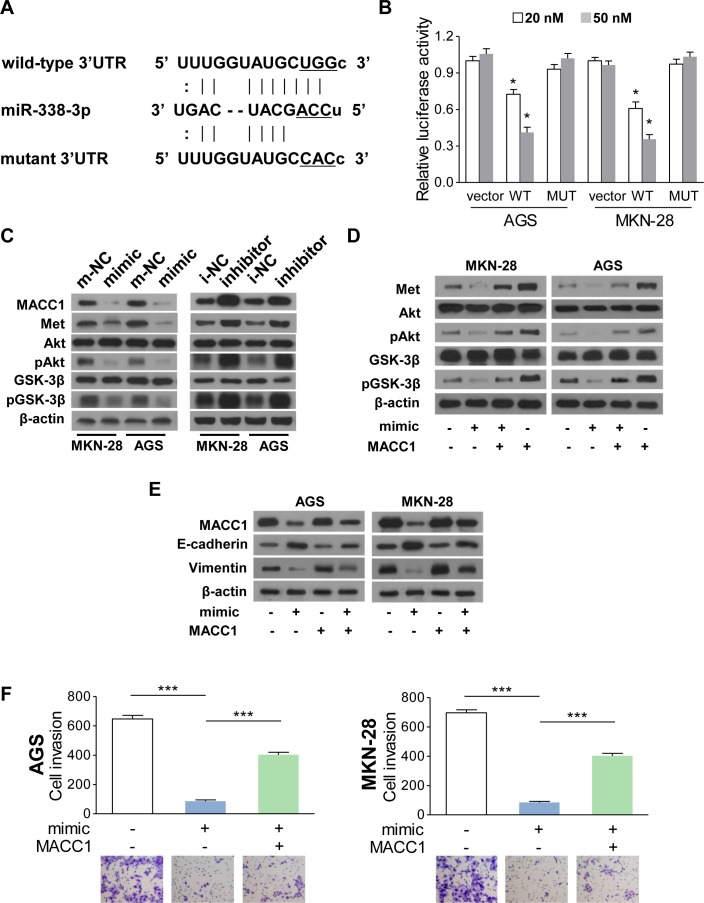
MiR-338-3p suppresses EMT via MACC1/Met/Akt signaling (**A**) MACC1 is predicted as the downstream target of miR-338-3p. The luciferase reporter of the miR-338-3p binding site on the wild-type and mutant MACC1 3′UTR. The replaced site is underlined. (**B**) Relative luciferase activity analyses. The vector, psiCHECK2-wMACC1, or psiCHECK2-mMACC1 plasmid (20 nM or 50 nM) was transfected into AGS and MKN-28 cells with or without miR-338-3p mimic. (**C**) MACC1, Met, pGSK-3β, GSK-3β, pAkt and Akt protein expression levels in AGS and MKN-28 cells transfected with miR-338-3p mimic, inhibitor, or the corresponding control plasmids. (**D**) Restoring MACC1 expression increased the protein expression of MACC1, Met, pGSK-3β, and pAkt in the miR-338-3p mimic-transfected cells (**E**) MiR-338-3p decreased MACC1 and vimentin expression and increased E-cadherin expression, whereas the reintroduction of MACC1 reversed these alterations. (**F**) Restoration of MACC1 re-enhanced miR-338-3p-dimished invasiveness in AGS and MKN-28 cells. **P < 0.05 and ***P < 0.001.

Our previous studies have shown that MACC1 promotes EMT progression in GC cells [15]. These findings made us curious of the relationship between miR-338-3p and MACC1 in GC. Searching for the potential binding sites of miR-338-3p (www.microrna.org), we found that the 3′UTR of MACC1 also contains the binding sequence of miR-338-3p, indicating that MACC1 might be another potential target gene of miR-338-3p (Figure 5A). To determine whether miR-338-3p could directly bind to the 3′UTR of MACC1, either wild-type or mutant 3′UTR MACC1 with a luciferase reporter gene (Figure 5A) was cotransfected with the miR-338-3p mimic into AGS and MKN-28 cells. Compared to the vector-transfected cells, luciferase activity was diminished in cells cotransfected with the miR-338-3p mimic and wild-type MACC1 3′UTR, whereas no significant decrease was observed in the mutant MACC1 3′UTR group (Figure 5B). Western blotting assay also showed that MACC1 protein levels were reduced in GC cells with miR-338-3p mimic and upregulated in cells with miR-338-3p inhibitor (Figure 5C). These findings demonstrated that miR-338-3p downregulated MACC1 expression by binding to its 3′UTR directly.

MACC1 transcriptionally regulates the expression of Met [19], which was reported to modulate the expression of EMT markers and induce EMT [20,21]. Therefore, we examined whether miR-338-3p could inhibit EMT by targeting the MACC1/Met signaling. Western blot analysis showed that, Met protein expression significantly decreased in GC cells transfected with miR-338-3p mimic, but increased and constitutively phosphorylated in the miR-338-3p-inhibited cells (Figure 5C and Figure S2). MiR-338-3p decreased Met and pMet (Tyr1234/1235) expression. Restoring MACC1 expression increased the protein expression of Met and pMet (Tyr1234/1235) in mimic transfected cells. In addition, previous studies have shown that Met signaling activates Akt and abrogates GSK-3β activity, which then induces the EMT process [20,22]. In the present study, we found that the levels of both phosphorylated Akt (pAkt) and phosphorylated GSK-3β (pGSK-3β) decreased in the miR-338-3p mimic GC cells but elevated in the miR-338-3p inhibited cells (Figure 5C). Moreover, restoring MACC1 expression counteracted the downregulation of pAkt and pGSK-3β, induced by miR-338-3p (Figure 5D).

To explore whether MACC1 cooperates with miR-338-3p in GC cell EMT modulation, we transduced MACC1-overexpressing plasmids together with the miR-338-3p mimic into GC cells. The expression of MACC1, E-cadherin, and vimentin was verified by western blot analysis. As shown in Figure 5E, MACC1 restoration decreased E-cadherin expression and increased vimentin expression by the miR-338-3p mimic. Moreover, the transwell invasive assay confirmed that reintroduction of MACC1 reinforced the cell invasiveness and migratory ability of GC cells, which were suppressed by the miR-338-3p mimic (Figure 5F).

Based on these findings, we concluded that miR-338-3p suppresses EMT by targeting the MACC1/Met/Akt pathway in GC cells.

### MiR-338-3p is inversely correlated with MACC1, ZEB2, and N-cadherin in GC tissues

**Figure 6:**
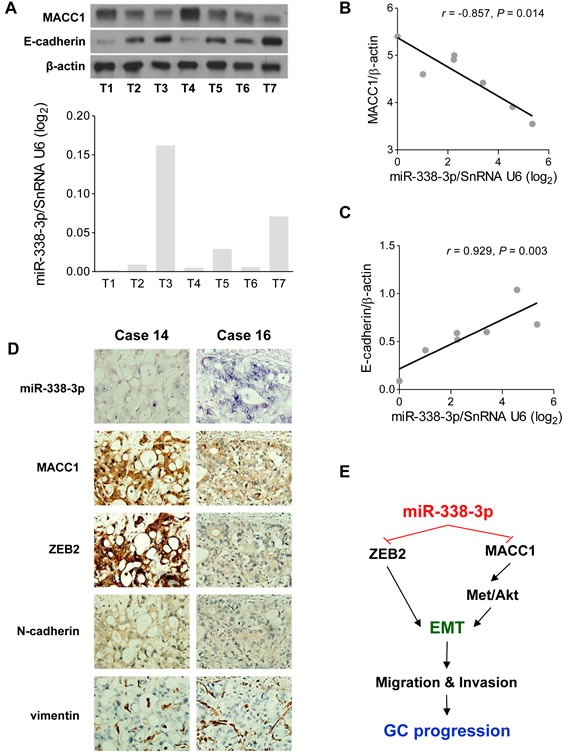
The EMT suppressive effect induced by miR-338-3p is confirmed in GC tissues (**A**, **B**, and **C**) Western blotting assay was performed in human GC tissues (n = 7). MiR-338-3p was inversely correlated with (B) MACC1 and (C) E-cadherin protein expression in GC tissues. (**D**) ISH study of miR-338-3p and IHC analysis of ZEB2, MACC1, N-cadherin, and vimentin was performed in 20 human GC tissues. MiR-338-3p expression was inversely associated with MACC1, ZEB2, and N-cadherin. Two representative cases are shown. (**E**) Schematic representation of this study. Both ZEB2 and MACC1 are directly targeted by miR-338-3p in GC. MACC1 suppression further inhibited its downstream Met/Akt signaling and suppressed pGSK-3β phosphorylation. These effects stabilized the epithelial marker, E-cadherin and inhibited the mesenchymal marker, N-cadherin and vimentin, which resulted in the inhibition of EMT. Therefore, the findings of the present study suggests that miR-338-3p is a microRNA against GC cell migration and invasion via its EMT modulating effect.

The aforementioned studies have shown that miR-338-3p negatively regulates EMT through MACC1 in GC cells. Next, we want to confirm the relationship between the expressions of miR-338-3p and MACC1 in human GC. The protein expression levels in human GC tissues were analyzed by Western blotting assay and half-quantified by the gray scale values (Figure 6A). Correspondingly, qRT-PCR was also conducted to determine the miR-338-3p levels in GC tissues from the same patient. The results showed that the MACC1 protein levels in GC tissues were inversely correlated with miR-338-3p mRNA expression levels (n = 7, r = −0.857, *P* = 0.014, Figure 6B). In these solid GC tissues, we investigated whether the epithelial biomarker E-cadherin was influenced by miR-338-3p. As anticipated, a significant correlation between miR-338-3p levels and E-cadherin protein levels was also observed in these GC tissues (n = 7, r = 0.929, *P* = 0.003, Figure 6A and 6C). Moreover, ISH was also performed to detected miR-338-3p expression, and IHC (immunohistochemistry) was performed to analyze MACC1, ZEB2, N-cadherin, and vimentin protein levels in 20 human GC tissues. We confirmed that MiR-338-3p expression was inversely associated with MACC1, ZEB2, and N-cadherin. No significance correlation in GC tissues was found between miR-338-3p and vimentin (Figure 6D), neither in MACC1 and ZEB2 (Figure S3). Taking this clinical result together with the findings of the aforementioned experimental studies, we conclude that miR-338-3p suppresses MACC1 and ZEB2 expression and helps to stabilize the epithelial phenotype in human GC.

## DISCUSSION

The present study verified that miR-338-3p is deficient in advanced GC. The in vitro experiments showed that restoration of miR-338-3p repressed the motility and invasiveness of GC cells. To explore the underlying mechanisms, we identified that miR-338-3p inhibited GC cell EMT progression by directly targeting ZEB2 and MACC1/Met/Akt signaling pathway. Finally, these molecular changes were also confirmed in human GC samples.

MiR-338-3p was originally identified to contribute to the basolateral polarity formation in the epithelial cells [23]. Its first report in cancer was the discovery of its invasion suppressive feature in hepatocellular carcinoma [11]. These studies suggested that miR-338-3p may participate in the EMT regulation, which requires cytoskeleton remodeling and motor activity. Therefore, we hypothesized that miR-338-3p inhibits GC progression by suppressing the EMT process.

As demonstrated by our clinical data, miR-338-3p expression is significantly reduced in Stage III and IV GC. Since Stages III and IV are greatly determined by the status of the N and M stage, which represents tumor invasiveness, we then performed functional experiments to verify the role of miR-338-3p in preventing GC cell invasion. These results were consistent with the findings of pervious studies that observed that miR-338-3p inhibited cancer cell invasion and metastasis [9,11]. However, the underlying mechanisms were unclear. As far as we know, this is the first study that focused on the mechanisms of miR-338-3p suppressing GC cell invasiveness via EMT inhibition. We also noticed that miR-338-5p is the other member of miR-338. It was reported that miR-338-5p upregulation was a marker for early-stage colon cancer and was upregulated with advanced TNM stages. Unlike its “brother” miR-338-5p, we observed that miR-338-3p is downregulateded in advanced GC. A recent report showed that miR-338 expression was reduced in GC, and miR-338 inhibited GC cell migration and invasion, as well as altered EMT marker expressions by directly targeting NRP1 [9]. Here, we provide clearer and more detailed information on the potential regulatory mechanism of miR-338-3p on the EMT process of GC cells by targeting EMT-associated transcription factors and integrating EMT signaling.

In the present study, we verified that the EMT transcription factor ZEB2 is one of the downstream targets of miR-338-3p. As previously reported, ZEB2 was highly expressed in the intestinal type GC [3] and was immunohistochemically positive in more than half of the GC tissues [4]. ZEB2 was shown to contribute to the loss of epithelial marker E-cadherin and disrupt cell-to-cell adhesion. In addition, ZEB2 was also reported to upregulate mesenchymal markers, which include N-cadherin and vimentin, as well as facilitate tumor cell invasion [8,24]. Our present study demonstrated that upregulating ZEB2 reverses the miR-338-3p-induced EMT inhibition, verifying that the EMT induced by miR-338-3p deficiency in GC requires ZEB2 participation. Previous studies indicated that miR-200b and miR-200c play an important role in EMT [17,18], and this is also confirmed in our study. As supplement to miR-200, our findings showed that miR-338-3p also act as an EMT inhibitor.

Our previous study demonstrated that MACC1 is an oncogene that indicates a poor prognosis in GC and induces EMT [15], as well as promotes the Warburg effect [25] and lymphangiogenesis [26]. In the present study, we also observed that MACC1 is directly inhibited by the binding of miR-338-3p to its 3′UTR, leading to the suppression of EMT. Taken these results together with that of miR-338-3p suppressing ZEB2, we believe that the effect of EMT inhibition by miR-338-3p is multiplex. MACC1 transcriptionally promotes Met expression and activates the HGF/Met pathway [19], whereas aberrant activating Met signaling causes Akt phosphorylation, which diminishes the activity of GSK-3β and decreases Snail phosphorylation, and consequently leads to EMT [22,27]. Since Met amplification may be a potential therapeutic target in GC [28], the result of present study imply the novel therapeutic value of miR-338-3p in GC by targeting MACC1/Met/Akt pathway.

In addition, several other pathways are currently known to contribute to cell invasiveness and motility, and these could be targeted by miR-338-3p. For example, smoothened, which instantiates hedgehog pathway, has been shown to be directly downregulated by miR-338-3p in liver cancer [11]. MiR-338-3p has also been reported to bind to PREX2a mRNA and further suppressed the PTEN/Akt pathway [29]. These findings indicate that there may be more than one channel for miR-338-3p to inhibit the EMT process. In this study, we believe that the MACC1/Met/Akt pathway is one of the decisive pathways, because when we introduced MACC1 into miR-338-3p-expressing cells, the EMT suppressive effect was dramatically reversed. Finally, our experimental results were further confirmed by our clinical data that miR-338-3p was negatively correlated with MACC1, ZEB2, and N-cadherin expression, and positively correlated with E-cadherin expression in human GC tissues. However, no significant correlation in GC tissues was found between MACC1 and ZEB2, indicating that ZEB2 and MACC1 induce biological effects via different mechanisms. This increased more confidence on our laboratory results that miR-338-3p changes influence the vast network of EMT.

In summary, the present study has provided further evidence that miR-338-3p suppresses the migration and invasion of GC cells and inhibits the EMT progress. In addition, ZEB2 and MACC1 were inhibited by having their 3′UTR directly bound by miR-338-3p. The MACC1/Met/Akt pathway is also involved in miR-338-3p-mediated EMT regulation (Figure 6E). Taken together, these findings reveal the novel EMT suppressing function of miR-338-3p, which might potentially become a novel strategy for targeting EMT.

## MATERIALS AND METHODS

### Tissue samples

The present study was approved by the Nanfang Hospital Research Ethics Committee, and informed consent was obtained from each patient. Tissue samples were obtained from the patients who underwent surgical resection at Nanfang Hospital, Southern Medical University. All fresh samples were immediately frozen after resection and stored at −80°C until use. Tumors are staged according to the TNM staging classification of stomach carcinoma of American Joint Committee on Cancer (AJCC, 7th ed., 2010).

### Cell lines and cell cultures

Human GC cell lines (AGS, MKN-28, NCI-N87, and MKN-45) and human gastric mucosa epithelial cells (GES-1) were propagated in RPMI1640 (Invitrogen, Carlsbad, CA, USA) supplemented with 10% fetal bovine serum [FBS (HyClone, Logan, UT, USA)]. HEK-293T cells were cultured in DMEM (Gibco-BRL) with 10% FBS.

### Plasmids and cell transfection

Cells were transfected with Lipofectamine 2000 Reagent (Invitrogen) following the manufacturer's protocol. A miR-338-3p mimic, miR-338-3p inhibitor or their corresponding controls (m-NC for mimic and i-NC for inhibitor) (Ribobio, Guangzhou, China) was used for transfection in this study.

For dual-luciferase reporter assay, the 3′UTR of ZEB2 that contained the wild-type or mutant putative binding site was inserted into the psiCHECK2 vector (Promega, Madison, WI). The same procedure was conducted for MACC1. The dual-luciferase reporter plasmid psiCHECK2-wMACC1 (containing the wild-type MACC1 putative 3′UTR binding site) and psiCHECK2-mMACC1 (contained mutant MACC1 3′UTR) were constructed. The primer sequences used for the dual-luciferase reporter plasmid are shown in the Supplementary Table 1.

For upregulated ZEB2 expression, GFP-tagged of human ZEB2, transcript variant 1 as transfection-ready DNA and a PrecisionShuttle mammalian vector with C-terminal tGFP tag were used (ORIGENE, Rockville, MD). AGS and MKN-28 cells were transfected according to the manufacturer's protocol. For MACC1 upregulation, the expression plasmid pBaBb-MACC1 was constructed as previously reported [21], and pBaBb-vector was used as control.

### RNA extraction and quantitative qRT-PCR

Total RNA from tissues and cells were extracted using the TRIZOL reagent (Invitrogen) and reverse transcribed by using the M-MLV RT kit (Promega). For the detection of mature miR-338-3p, specific RT primers and PCR primers (Ribobio) were constructed, and cDNA was amplified using SYBR^®^ Green PCR Master Mix (Toyobo, Osaka, Japan). The relative transcript levels of E-cadherin, N-cadherin, fibronectin, vimentin and ZEB2 were detected using SYBR^®^ Green qPCR SuperMix (Invitrogen). The primers sequences are listed in the Supplementary Table 2. Relative expression was normalized to *snRNA U6* or *β-actin* and calculated using the 2^−ΔΔCt^ method.

### ISH and IHC analysis

ISH was performed on paraffin-embedded tissues sections containing 20 pairs of GC tissues and corresponding non-cancerous stomach tissue. The miR-338-3p probes were double digoxigenin (DIG)-labeled mercury locked nucleic acid probes [miRCURY LNA^TM^ detection probes (Exiqon, Vedbaek, Denmark)]. The sequences were as follows: 5′-CAA CAA AAT CAC TGA TGC TGG A-3′. IHC procedures were conducted in 20 GC tissues samples as previously described [15,25]. The following primary antibodies were used: anti-ZEB2, anti-N-cadherin, and anti-vimentin (Abcam); anti-MACC1 (ImmunoWay, Newark, DE, USA). Staining patterns were evaluated by two independent reviewers, and the half-quantitative scoring system was as previously described [15, 25].

### Migration and invasion assays

For the migration assay, wound healing assay was performed in 6-well plates. After 48 h of transfection, streaks were created by scraping the confluent cell monolayer with a 200 μL sterile pipette tip and washing twice with PBS. The migration of cells was observed at 0 h and 48 h after wounding and then photographed. The relative migration rate of the cells was calculated based on the width at the 0 h time point.

For the invasion assay, at 48h after transfection, 1.0 × 10^5^ cells in serum-free medium were seeded into the upper chamber (Corning, NY, USA) that was pre-coated with Matrigel (BD Biosciences, CA, USA). The lower chamber was filled with 500 μL of RPMI 1640 supplemented with 10% FBS as a chemoattractant. After incubating for 24 h at 37 °C with 5% CO_2_, non-invading cells on the upper surface of the membrane were scrubbed by cotton swabs. Cells adhering to the lower membrane were fixed with 4% paraformaldehyde, stained with crystal violet solution, and then counted in 10 random fields per well under microscope.

### 3D cell culture

Forty-eight hours after transfection, cells (density: 2 × 10^4^) were trypsinized and plated onto 24-well plates with pre-coated Matrigel (BD Biosciences) following the methods that had been used in our previous studies [15]. The culture medium was replaced every three days. After 12 days of culturing, the cells were photographed.

### Western blot analysis

Cells and tissues were lysed on ice in RIPA lysis buffer supplemented with protease inhibitor (Beyotime, Shanghai, China). Protein expressions was detected by western blot analysis as previously described [25]. The following primary antibodies were used: anti-E-cadherin, anti-N-cadherin, anti-vimentin, anti-Met, anti-pMet (Try1003, Try1234/1235 and Try1349), anti-Akt, anti-pAkt, anti-GSK-3β and anti-pGSK-3β (Cell Signaling Technology, Danvers, MA); anti-fibronectin and anti-ZEB2 (Abcam, Cambrige, MA); anti-MACC1 (Abnova, Taipei, China), β-actin (Boster, Wuhan, China) and HRP-conjugated anti-GAPDH (Kangchen, Shanghai, China). Protein signals were developed after incubation with HPR-conjugated goat-anti-rabbit IgG (SouthernBiotech, Birmingham, AL) or goat-anti-mouse IgG (Boster) secondary antibody by using the chemiluminescence (ECL) system. The expression level of MACC1 protein in the GC tissues was half-quantified with Image J software.

### Dual-luciferase reporter assay

To determine whether miR-338-3p targeted ZEB2 3′UTR, dual-luciferase activity assays were performed as previously described [30]. For MACC1 3′UTR reporter analysis, GC cells AGS and MKN-28 were seeded in a 24-well plate one day before transfection. MiR-338-3p mimics (50 nM) and 0.5 μg of psiCHECK2 report plasmids (psiCHECK2-wMACC1 or psiCHECK2-mMACC1) were cotransfected into GC cells using the Lipofectamine 2000 reagent. At 48 h after transfection, luciferase activities were measured using the dual-luciferase reporter assay system (Promega). Transfections were performed in triplicate.

### Statistical analysis

All statistical analyses were performed using the SPSS13.0 software (SPSS Inc., Chicago, IL), and data were expressed as the mean ± SEM. *P* < 0.05 was considered statistically significant. MiR-338-3p expression in advanced-stage GC patients and early-stage patients was analyzed using the Mann-Whitney U test. For cell line experiments, data were subjected to a two-tailed Student t test or one-way ANOVA (T test for 2-group comparisons, otherwise one-way ANOVA). The correlation between miR-338-3p and ZEB2, MACC1, E-cadherin, N-cadherin, and vimentin was determined by Spearman's rank test. The experiments were repeated at least in triplicate.

## SUPPLEMENTARY MATERIAL TABLES AND FIGURES


