# Supplementary material for: MiR-338-3p inhibits epithelial-mesenchymal transition in gastric cancer cells by targeting ZEB2 and MACC1/Met/Akt signaling
Source: Oncotarget. 2015 Apr 14;6(17):15222–34. doi: 10.18632/oncotarget.3835 (PMC4558147; doi:10.18632/oncotarget.3835)
Supplement: Supplementary file 1 [file oncotarget-06-15222-s001.pdf]

MiR-338-3p inhibits epithelial-mesenchymal transition in gastric cancer cells by targeting ZEB2 and MACC1/Met/Akt signaling

Supplementary Material

Supplementary Table 1

The sequences of primer used for dual-luciferase reporter plasmid.

Primer	Forward (5′-3′)	Reverse (5′-3′)
**ZEB2**	TAAACTACTGCATTTTAAGCTTCCTATTT	ACTGAAAAACTGTCTTAGAAAATCACG
**Mut ZEB2**	AATTTCTTTTGAAAATGACC*TACGAGC*AATAAAAATGTAGCCAAACT	AGTTTGGCTACATTTTTATT*GCTCGTA*GGTCATTTTCAAAAGAAATT
**MACC1**	CCGCTCGAGGGAGCTGAATAGGTGCAGAT	ATAAGAATGCGGCCGCCTGCCTCAGCCTCCTGAGTA
**Mut MACC1**	CCACCCTGGGATATTTGGTATGC*CAC*CAATGGTAATTCAAATT	AATTTGAATTACCATTG*GTG*GCATACCAAATATCCCAGGGTGG

Supplementary Table 2

The sequences of primer used for qRT-PCR.

Primer	Forward (5' -3')	Reverse (5' -3')
**E-cadherin**	CTGCTGCAGGTCTCCTCTTG	TGTCGACCGGTGCAATCTTC
**N-cadherin**	ACAGTGGCCACCTACAAAGG	CCGAGATGGGGTTGATAATG
**Fibronectin**	GACCACATCGAGCGGATCTG	GTCTCTTGGCAGCTGACTCC
**Vimentin**	AAGGCGAGGAGAGCAGGATT	GGTCATCGTGATGCTGAGAAG
**ZEB2**	CTCTTCCCACACGCTTAGTT	GGCCTAAGCTTACAGTGTCATG
**β-actin**	TGGATCAGCAAGCAGGAGTA	TCGGCCACATTGTGAACTTT

Supplementary Results

Supplementary Table 3

Clinicopathological characteristics of GC patients

Case	Age	Gender	Histological differentiation	Clinical stage	T classification	N classification	Metastasis
**1**	74	Male	Moderately	IV	T4b	N1	Yes
**2**	53	Male	Poorly	IIIC	T4b	N3	No
**3**	71	Male	Moderately	IIB	T4a	N0	No
**4**	55	Male	Poorly	IV	T4a	N1	Yes
**5**	69	Male	Poorly	IIA	T2	N1	No
**6**	44	Male	Poorly	IIIB	T4a	N2	No
**7**	62	Female	Well	IA	T1b	N0	No
**8**	46	Male	Poorly	IIA	T1b	N2	No
**9**	70	Male	Poorly	IIB	T2	N2	No

**10**	79	Male	Poorly	IB	T2	N0	No
**11**	41	Male	Poorly	IA	T1b	N0	No
**12**	56	Male	Well	IB	T2	N0	No
**13**	77	Male	Poorly	IIIC	T4a	N3	No
**14**	57	Male	Poorly	IIIB	T4b	N1	No
**15**	73	Male	Moderately	IIIA	T4a	N1	No
**16**	51	Female	Poorly	IIIA	T4a	N1	No
**17**	61	Male	Moderately	IIA	T2	N1	No
**18**	58	Female	Poorly	IIIB	T4a	N2	No
**19**	78	Male	Moderately	IIIC	T4b	N2	No
**20**	48	Male	Poorly	IIIA	T4a	N1	No

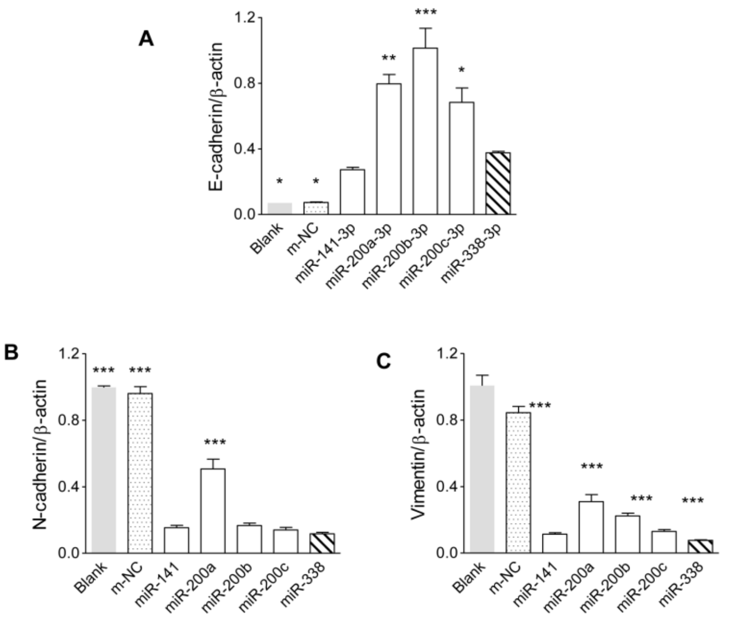

Figure S1. E-cadherin, N-cadherin, and vimentin mRNA expression levels in MKN-28 cells transfected with miR-200 family (miR-200a-3p, miR-200b-3p, miR-200c-3p and miR-141-3p) mimics or miR-338-3p mimics by qRT-PCR.

(A) Compared with miR-338-3p, the expression of E-cadherin mRNA was significantly increased in MKN-28 cells transfected with miR-200b-3p, miR-200a-3p, or miR-200c-3p. **(B** and **C)** N-cadherin and vimentin mRNA levels in miR-338-3p-transfected cells were lower than the levels in miR-200a-3p-transfected cells.

*$P < 0.05$, **$P < 0.01$ and ***$P < 0.001$.

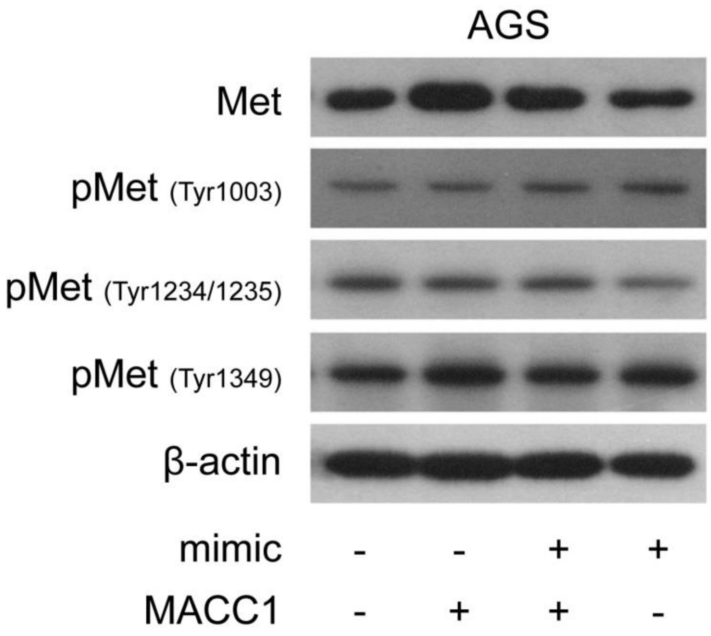

Figure S2. Western blot analysis of MACC1, Met and p-Met protein expression in AGS cells.

MiR-338-3p decreased Met and pMet (Tyr1234/1235) expression. Restoring MACC1 expression increased the protein expression of Met and pMet (Tyr1234/1235) in mimic transfected cells.

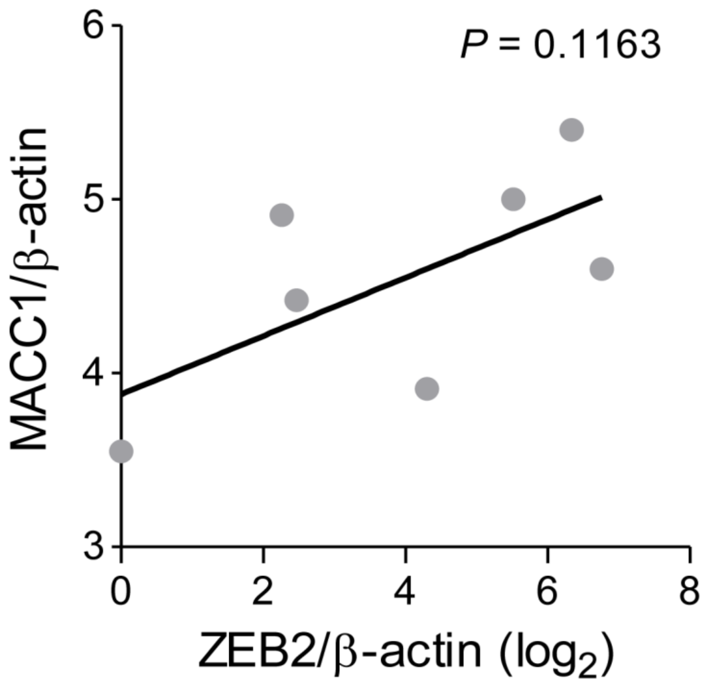

Figure S3. No siginificance correlation was found between MACC1 and ZEB2.
